# Impact of Barrett oesophagus diagnoses and endoscopies on oesophageal cancer survival in the UK: A cohort study

**DOI:** 10.1002/cam4.4484

**Published:** 2021-12-16

**Authors:** Judith Offman, Francesca Pesola, Rebecca C. Fitzgerald, Willie Hamilton, Peter Sasieni

**Affiliations:** ^1^ Comprehensive Cancer Centre School of Cancer and Pharmaceutical Sciences Faculty of Life Sciences & Medicine King’s College London London UK; ^2^ Current affiliation: Health and Lifestyle Research Unit Wolfson Institute of Preventive Medicine Queen Mary University of London London UK; ^3^ MRC Cancer Unit Hutchison/MRC Research Centre University of Cambridge Cambridge UK; ^4^ College of Medicine and Health University of Exeter Exeter UK

**Keywords:** Barrett oesophagus, lead‐time bias, oesophageal cancer, relative survival, surveillance

## Abstract

**Background:**

Current guidelines recommend endoscopic surveillance for Barrett oesophagus (BE), but the value of surveillance is still debated. Using a combination of primary care, secondary care and cancer registry datasets, we examined the impact of a prior BE diagnosis, clinical and risk factors on survival from oesophageal cancer and adenocarcinoma.

**Methods:**

Retrospective cohort study of patients aged 50 and above diagnosed with malignant oesophageal cancer between 1993 and 2014 using Clinical Practice Research Datalink (CPRD). All prior BE diagnoses and endoscopies were identified from CPRD and Hospital Episode Statistics. Histology information was obtained from linked cancer registry data. We used flexible parametric models to estimate excess hazard ratios (EHRs) for relative survival. We simulated the potential impact of lead‐time by adding random lead‐times from a variety of distributions to all those with prior BE.

**Results:**

Among our oesophageal cancer (*n* = 7503) and adenocarcinoma (*n* = 1476) cohorts only small percentages, 3.4% and 5.3%, respectively, had a prior BE diagnosis. Two‐year relative survival was better among patients with BE: 48.0% (95% CI 41.9–54.9) compared to 25.2% (24.3–26.2) without. Patients with BE had a better prognosis (EHR = 0.53, 0.41–0.68). Survival was higher even if patients with BE had fewer than two endoscopies (50.0%; 43.6–57.3). A survival benefit was still observed after lead‐time adjustment, with a 20% absolute difference in 2‐year survival using a 5 year mean sojourn time.

**Conclusions:**

Patients with a prior BE diagnosis had a survival advantage. This was not fully explained by surveillance endoscopies.

## Graphical abstract

With the value of surveillance for Barrett oesophagus (BE) is still being under debate, we examined the impact of a prior BE diagnosis on survival from oesophageal cancer using a novel method for lead‐time adjustment. A prior BE diagnosis conferred a survival benefit, which was still observed after lead‐time adjustment, and survival was higher even if no or only one surveillance endoscopy was carried out.

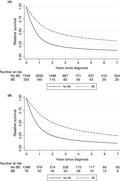

## 1 INTRODUCTION

The incidence of oesophageal cancer (EC) has increased dramatically in the developed world over the last 30 years and it is now the 8th most common cancer worldwide.^1^ The majority of ECs occur as either squamous cell carcinomas or oesophageal adenocarcinomas (EACs).^2^ EAC generally originates from Barrett oesophagus (BE), a complication of chronic gastro‐oesophageal reflux disease (GERD). As screening all GERD patients for BE using endoscopy is not feasible,^3^ most EACs present *de novo* without a prior diagnosis of BE.^4^ EAC carries a poor prognosis despite advances in neoadjuvant therapy and surgery.^5^ A number of factors that modify the risk for EAC have been reported. The two strongest risk factors for EAC are GERD and obesity.^6^, ^7^ In addition, tobacco smoking is a moderately strong risk factor.^8^ The association between socioeconomic status and risk of EAC is currently not clear, with contradicting studies suggesting an increase, decrease or no effect of higher socioeconomic status (SES).^9^ A number of observational studies have suggested that regular acid suppressant treatment with proton pump inhibitors (PPIs), like Esomeprazole, could reduce the risk of neoplastic progression in patients with BE.^10^ The AspECT trial furthermore showed that high dose PPI taken for more than 10 years delayed diagnosis of cancer, high‐grade dysplasia and death in patients with BE.^11^ This benefit was, however, mostly driven by improved all‐cause mortality rather than a reduced cancer diagnosis.

Current guidelines from the British Society of Gastroenterology recommend 3–5 yearly endoscopic surveillance for BE without dysplasia shorter than 3 cm, and 2–3 yearly surveillance for segments of 3 cm or longer.^3^ However, the value of surveillance endoscopy for patients with BE is still debated, as inconsistent results have been reported for the impact of these surveillance strategies. A recent study found that EAC patients with evidence of BE at the time of their cancer diagnosis, but without prior surveillance endoscopies had increased survival compared to patients without evidence of BE.^12^

This is the first study to investigate EC and EAC survival across a comprehensive national health care database (NHS) in the United Kingdom (UK). We used a combination of primary care, secondary care and cancer registry datasets to identify a large UK oesophageal cancer cohort of over 7000 patients diagnosed between 1993 and 2014 with the aim of examining the impact of a prior Barrett's oesophagus diagnosis, clinical factors and risk factors on survival from oesophageal cancer and adenocarcinoma. As previous studies only examined patients with a prior BE diagnosis or only BE patients in surveillance programmes, without taking into consideration the potential interplay between the presence of BE and the number of surveillance endoscopies, we wanted to examine the impact of regular surveillance amongst patients with BE. Lastly, we carried out a series of sensitivity analyses to adjust for lead‐time. Although these individuals could not all be assumed to have surveillance‐detected cancers, it is reasonable to expect that they would have been more likely to have had surveillance for existing BE and therefore some would have had their cancers diagnosed earlier. We, therefore, used a mixture distribution for lead‐time with 50% of subjects presumed to have no lead‐time. As the sojourn time for EC is not known we considered a range of sojourn times from 2 to 7 years and applied these to the 50% of subjects assumed to have surveillance‐detected cancers. As histology was only available for about one‐third of these patients, we analysed both the entire EC cohort and the EAC to ensure complete ascertainment of all tumours with a prior BE diagnosis.

## 2 METHODS

### 2.1 Study population and design

The Clinical Practice Research Datalink (CPRD) is a primary care database covering over 11.3 million patients from 674 UK practices.^13^ 75% of all English (58% of all UK) CPRD practices are linked to Hospital Episode Statistics (HES) for hospital data, Office of National Statistics (ONS) for mortality data, Index of Multiple Deprivation (IMD), and National Cancer Registration and Analysis Service (NCRAS) for cancer registry data.^13^

We carried out a retrospective cohort study using CPRD data, linked to NCRAS, for morphology and site data, HES to obtain data on endoscopies and BE diagnosis, and IMD. All patients diagnosed with malignant EC between January 1993 and December 2014 were identified using EC specific Read codes (see Table S1). The date of diagnosis, that was used to enter patients into the cohort, used the first record of an EC diagnosis in the CPRD dataset, which would have been based on the diagnostic endoscopy. Due to diagnoses in CPRD, which had been originally made in secondary care, having been recorded at a later date following letters from hospital clinics, there might have been a discrepancy of 0–20 between the date recorded in CPRD and by NCRAS in most cases.^14^ Other inclusion criteria were age 50+ at EC diagnosis, at least 3 years CPRD registration prior to EC diagnosis and a valid date of death from either ONS or CPRD. Linkages were available for 59% (*n* = 3920) of EC patients. Linked data from NCRAS was available from 1992 to 2010, whereas linked HES and ONS mortality data was available from April 1997 to December 2013 and January 1998 to December 2013, respectively. CPRD records do not include information on cancer morphology and stage, so to identify EACs morphology data was obtained from NCRAS data using International Classification of Diseases for Oncology version 3 (ICD‐O‐3) codes.

Date of entry into the study cohort was defined as the first CPRD record of EC diagnosis. Patient records either up to 15 years prior to their EC diagnosis, or their patient registration date, if it was less than 15 years earlier, were examined for records of upper GI diagnoses and symptoms, endoscopies, acid suppressant medication and demographic covariates (BMI and smoking). All BE diagnoses (for Read codes see Table S2), other recorded upper GI symptoms or diagnoses, endoscopies and demographic information were extracted from the CPRD dataset using Read codes. All PPI and H2 receptor antagonist (H2RA) prescriptions were identified and extracted using product codes. Additional BE diagnoses were identified from HES using International Statistical Classification of Diseases and Related Health Problems 10th revision (ICD10) codes. As the ICD10 code for BE has only been available since the 2006 version, additional BE cases could only be identified from 2006 onwards. BE diagnoses occurring up to 6 months before an EC diagnosis were considered to be prevalent cases and thus not included as prior BE diagnoses. Additional endoscopies were identified from HES admitted patient care data using Classification of Surgical Operations and Procedures 4th revision (OPCS‐4) codes. Any endoscopies recorded up to 6 months before the date of cancer diagnosis were excluded, as the cancer was likely to have been diagnosed as part of this procedure (i.e. diagnostic endoscopy). Endoscopy procedures that occurred during the same hospitalisation or within 30 days of each other were counted as one endoscopy episode. Date of death was obtained from ONS, where linked ONS data were available, or from CPRD for patients without linked ONS data. Number of consultations within one year prior to EC diagnosis were determined by the number of consultations in primary care. The time intervals used to categorise year of diagnosis were not linked to any changes in clinical practice but even time intervals were picked.

### 2.2 Statistical methods

Date of EC diagnosis was based on the date recorded in the CPRD or HES dataset. Frequencies and distribution of patients’ characteristics were calculated for all EC cancers and for EAC cancers only. Additionally, they were calculated separately for patients with and without pre‐cancer BE diagnosis.

As EC prognosis is poor, with only 16% of patients surviving 5 years,^15^ we were interested in understanding the impact of several factors on survival for 7 years after diagnosis. Due to the small number of patients at risk in the older age groups and potential presence of comorbidities, which cause death, patients <85 years were followed up to maximum age of 87 while patients aged ≥85 were only followed for a maximum of 2 years. This should avoid bias and instability. Patients were therefore followed from the date of diagnosis until either death, end of the follow‐up period (7 years, up to age 87 in 80+ or 2 years in ≥85) or end of study (31 December 2014), whichever occurred first.

#### 2.2.1 Relative survival

Relative survival is a measure of excess mortality and is calculated as the observed survival rates in EC (EAC) patients divided by the expected rates in the age‐, sex‐ and year‐matched general population.^16^ Expected survival was obtained from the UK Human Mortality Database^17^ life tables for the EC cohort, whereas life tables for England from the National Life Tables by ONS^18^ were used for the EAC subgroup.

We used flexible parametric models (FPM) to explore the impact of a number of known risk factors. We estimated excess hazard ratios (EHRs) and 95% confidence intervals (CI), where EHR values >1 indicate the presence of excess mortality (i.e. worse prognosis).^19^ The logarithm of the cumulative baseline excess hazard function was modelled using restricted cubic splines with 4 degrees of freedom. In the FPM framework relative survival is based on an extension of Royston‐Parmar.^20^, ^21^

The proportionality assumption of the various exposure variables was assessed by comparing the model fit for a model which assumed the effect to be constant over time with an alternative model where this assumption was relaxed. Model fits were compared using the likelihood ratio test. If the likelihood ratio test showed that relaxing the proportionality assumption improved the model fit, the effect was allowed to vary over time. We performed separate models for each factor adjusting for age and year of diagnosis (‘univariate’ model), as it is known that EC survival has improved over time since 1990s^22^ and that survival decreases with age.^15^ We also conducted a final multivariate model where all risk factors were included simultaneously to identify the strongest predictors of survival. For covariates with missing data, a missing data category was included in all models.

Based on the multivariate FPM model, we estimated 2‐year relative survival for each risk/protective factor while adjusting for age and year of diagnosis. Median survival for the overall sample and by BE diagnosis was obtained using an FPM model also adjusted for year of diagnosis and age using the STPM2_standsurv command.^23^

#### 2.2.2 Lead‐time adjustment

We conducted a series of sensitivity analyses correcting our survival analysis for potential lead‐time bias. The lead‐time adjustment used here is based on the method described by Massat et al.^24^ Lead‐time is the amount of time by which the date of diagnosis is advanced by BE surveillance compared to symptomatic detection. This is added to the time from counterfactual systematic diagnosis to death for patients with cancers detected by surveillance endoscopy and results in a survival bias in favour of surveillance. Sojourn time is the time period from when cancer is non‐symptomatic but detectable by endoscopy to symptomatic diagnosis. Lead‐time (t) was therefore estimated assuming an exponential distribution. As the sojourn time is not known and is unlikely to be homogeneous, we estimated the impact of a range of mean sojourn times from 2 to 7 years. The individual lead‐time (t) for the non‐symptomatic but endoscopy detectable phase was therefore estimated for each surveillance‐detected cancer assuming an exponential distribution, whereby for the rate of transition to symptomatic disease we used a range from (mean sojourn time = 2 years) to (mean sojourn time = 7 years). As in clinical practice, the frequency of surveillance endoscopy varies, the actual obtained lead‐time would vary as well. We, therefore, sampled an unconditional random variable from the exponential distribution of lead‐time ranging from zero to the maximum sojourn time separately for each sojourn time. We used a truncated exponential distribution to avoid allocating a longer lead‐time than the time between diagnosis and death in patients who died. Lastly, since we did not know the mode of detection (surveillance or symptomatic), but it was reasonable to assume that some were more likely to be diagnosed earlier due to surveillance, we used a mixture distribution for lead‐time with 50% having no lead‐time and 50% having a truncated exponential random lead‐time as described above.

## 3 RESULTS

### 3.1 Study cohort

A total of 7503 EC patients in the UK CPRD database met our inclusion criteria (Figure S1). Histology records were available for 2727 cases (36.4% of the total sample). Of these, 1476 (54.1%) patients had been recorded as having EAC, 875 (32.1%) SCC and 376 (13.8%) unspecified. The main focus of the paper is the overall EC cohort and subgroup of patients with EAC. Patient characteristics can be found in Table 1 and Table S3. The median age at cancer diagnosis was 72 years (interquartile range (IQR) = 64–80) in the EC cohort and the EAC subsample (IQR = 64–79). Among EC patients, only 255 (3.4%) had a prior BE diagnosis and 473 (6.3%) attended at least 2 endoscopy examinations. This proportion was slightly higher for the EAC subgroup, where 78 (5.3%) had a BE diagnosis and 140 (9.5%) patients had undergone 2+ endoscopies.

**TABLE 1:** Baseline characteristics of oesophageal cancer by BE diagnosis

Characteristic	No BE	BE
	*n* = 7248	*n* = 255
Age at cancer diagnosis (years)		
Median (IQR)	72 (64–80)	72 (64–79)
Sex, n (%)		
Male	4766 (66)	202 (79)
Female	2482 (34)	53 (21)
BMI, n (%)		
Underweight	512 (7)	17 (7)
Normal	2309 (32)	90 (35)
Overweight	1598 (22)	81 (32)
Obese	708 (10)	27 (11)
Missing	2121 (29)	40 (16)
Smoking, n (%)		
Current	2062 (29)	58 (23)
Never	2888 (40)	115 (45)
Ex	1730 (24)	78 (31)
Missing	568 (8)	4 (2)
IMD categories, n (%)		
1 (most deprived)	1308 (18)	53 (21)
2	1278 (18)	33 (13)
3	1450 (20)	57 (22)
4	1653 (23)	49 (19)
5 (least deprived)	1559 (22)	63 (25)
Clinical characteristics		
Morphology		
AC	1398 (19)	78 (31)
SCC	870 (12)	5 (2)
Other	369 (5)	7 (3)
Missing	4611 (64)	165 (65)
Prior endoscopies, n (%)		
None	3019 (42)	73 (29)
1	3802 (52)	136 (53)
2+	427 (6)	46 (18)
Number of years with 6+ PPI / H2RA, n (%)		
0	5307 (73)	9 (4)
1–3	776 (11)	29 (11)
4–6	512 (7)	56 (22)
7–9	312 (4)	56 (22)
10+	341 (5)	105 (41)
Most severe upper GI diagnosis prior to cancer diagnosis, n (%)		
No prior diagnosis	3923 (54)	47 (18)
Indigestion/reflux	2161 (30)	61 (24)
Ulcer	54 (0.8)	8 (3)
Oesophagitis	277 (4)	21 (8)
Hiatus hernia	486 (7)	102 (40)
Strictures	347 (5)	16 (6)
Year of diagnosis, n (%)		
1993–1999	904 (13)	2 (0.8)
2000–2006	2552 (35)	86 (34)
2007–2013	3792 (52)	167 (66)
Number of primary care consultation within one year prior to diagnosis, n (%)		
0–7	2876 (40)	87 (34)
8–14	2487 (34)	86 (34)
≥15	1885 (26)	82 (32)

Abbreviations: AC, adenocarcinoma; BE, Barrett oesophagus; H2RA, H2 receptor antagonist; PPI, proton pump inhibitor; SCC, squamous cell carcinoma.

BMI: Underweight: <18.5; Normal: 18.5–<25; Overweight: 25–<30; Obese ≥30.

### 3.2 Survival analysis

Overall, during the study period, there were 6407 (85.4%) deaths in the EC cohort (*n* = 7503). Of these 1335 (deaths occurred in the EAC subgroup (*n* = 1476; 90.4%)). The median survival was 8.5 (IQR = 3.2 to 22.3) months for all EC patients and 9.2 (IQR = 3.6 to 23.5) for the EAC subgroup. In the EC cohort, the median survival among BE patients was 20.2 (IQR = 7.4 to 71.3) months compared to 8.3 (IQR = 3.1 to 21.3) for patients without prior to BE diagnosis. In the EAC subgroup, the median survival for BE patients was 21.3 months (IQR = 8.1 to 59.1) compared to 8.8 (IQR = 3.5 to 22.1) for patients without BE.

**FIGURE 1:**
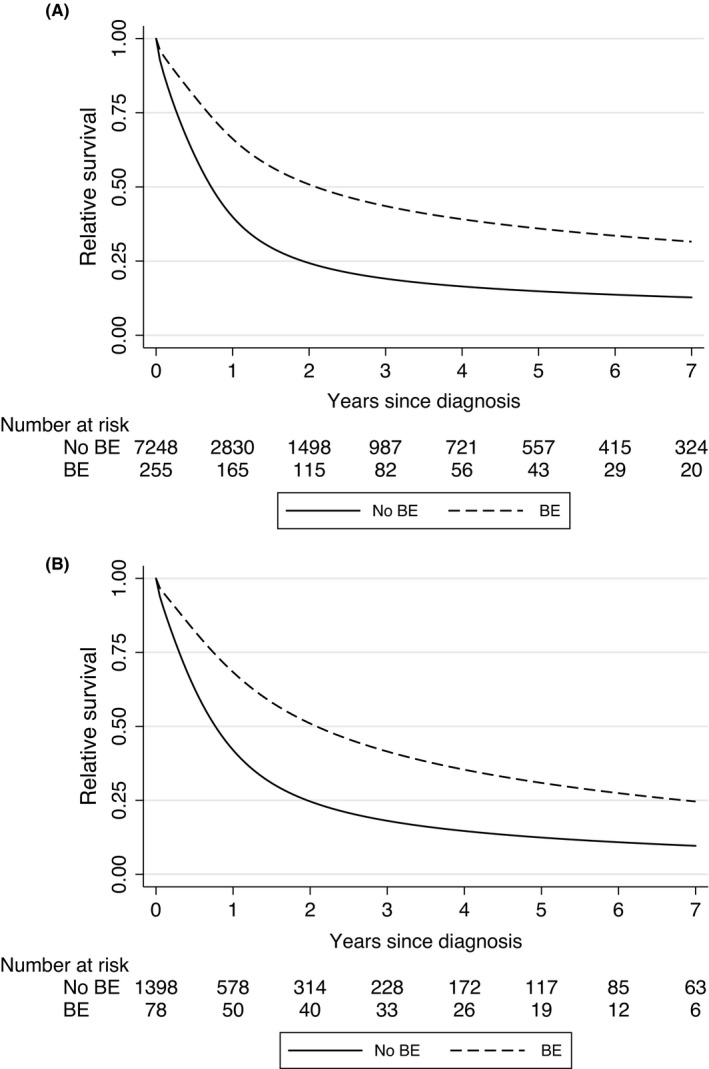
Flexible parametric survival model curves comparing survival for EC (A) and EAC (B) with or without prior BE diagnosis adjusted for age and year of diagnosis. Note that the curves are not forced to have proportional hazards. BE, Barrett oesophagus

The estimated 2‐year relative survival adjusting from the multivariate model showed that EC and EAC patients with a BE diagnosis had better survival than those without a BE diagnosis (Table 2); 2‐year relative survival for patients with and without prior BE was 48.0% (95% CI 41.9–54.9) and 25.2% (24.3–26.2) respectively for the whole EC cohort and 43.7% (95% CI 33.8–56.5) compared to 25.3% (23.3–27.4) for EAC. In addition, having at least two endoscopies before diagnosis was associated with improved survival: 32.4% (28.8–36.5) compared to 25.6% (24.3–27.1) with no endoscopy record for all ECs and 35.2% (28.7–43.2) compared to 20.9% (16.2–26.8) for the EAC subgroup. Figure 1 shows FPM survival curves for the predictor prior BE diagnosis.

**TABLE 2:** 2‐year relative survival and excess hazard ratio (EHR) for relative survival for all EC and EAC patients obtained using flexible parametric models for the whole observation period

Characteristic	EC (n = 7503)			EAC (n = 1472)		
	2‐year relative survival (95% CI)^a^	Multivariable model^b^		2‐year relative survival (95% CI)^a^	Multivariable model^b^	
	%	EHR (95% CI)	*p* value	%	EHR (95% CI)	*p* value
Age at diagnosis						
<65	36.1 (34.2–38.1)	1		36.7 (32.8–41.0)	1	
65–69	30.1 (28.3–33.2)	1.24 (1.11–1.38)	<0.001	31.2 (26.4–36.9)	1.17 (0.97–1.41)	0.095
70–74	28.4 (26.2–30.8)	1.36 (1.22–1.51)	<0.001	28.3 (23.7–33.7)	1.28 (1.06–1.54)	0.010
75–79	23.4 (21.3–25.6)	1.61 (1.46–1.79)	<0.001	21.3 (17.4–26.1)	1.59 (1.32–1.90)	<0.001
80–84	17.3 (15.3–19.6)	1.91 (1.72–2.13)	<0.001	15.7 (11.8–20.8)	1.93 (1.57–2.38)	<0.001
85–89	10.4 (8.2–13.1)	2.68 (2.38–3.02)	<0.001	9.9 (6.4–15.3)	2.47 (1.93–3.16)	<0.001
≤90	10.5 (7.3–15.0)	2.81 (2.40–3.28)	<0.001	9.8 (4.7–20.8)	2.48 (1.69–3.62)	<0.001
Sex						
Male	25.4 (24.3–26.5)	1		26.4 (24.3–28.8)	1	
Female	27.2 (25.7–28.9)	0.95 (0.89–1.01)	0.111	25.5 (21.8–30.0)	1.03 (0.89–1.19)	0.694
BMI category						
Underweight	25.7 (22.7–29.2)	1.08 (0.95–1.23)	0.213	34.5 (25.8–46.2)	0.93 (0.67–1.28)	0.652
Normal	28.7 (27.2–30.4)	1		32.1 (28.5–36.2)	1	
Overweight/obese	23.9 (22.5–25.5)	1.16 (1.07–1.26)	<0.001	21.3 (18.5–24.6)	1.41 (1.21–1.64)	<0.001
Missing	25.3 (23.6–27.1)	1.12 (1.03–1.22)	0.006	25.1 (21.9–28.8)	1.24 (1.06–1.45)	0.007
Smoking						
Current	21.4 (19.9–22.9)	1.27 (1.17–1.37)	<0.001	24.6 (21.0–28.7)	1.13 (0.97–1.32)	0.111
Never	29.7 (28.2–31.2)	1		28.5 (25.6–31.8)	1	
Ex	28.7 (26.9–30.7)	0.99 (0.91–1.08)	0.865	28.2 (24.6–32.3)	1.01 (0.87–1.17)	0.875
Missing	15.6 (12.9–18.8)	1.62 (1.45–1.82)	<0.001	10.7 (6.9–16.4)	1.93 (1.52–2.44)	<0.001
IMD Category						
1 (most deprived)	28.2 (26.2–30.4)	1		30.9 (26.4–36.1)	1	
2	26.5 (24.5–28.7)	1.05 (0.96–1.15)	0.258	27.1 (23.2–31.8)	1.12 (0.92–1.37)	0.245
3	25.6 (23.7–27.5)	1.09 (1.00–1.18)	0.060	26.9 (23.2–31.3)	1.13 (0.93–1.37)	0.208
4	25.6 (23.8–27.4)	1.09 (1.00–1.18)	0.540	24.5 (20.9–28.7)	1.22 (1.01–1.48)	0.040
5 (least deprived)	24.6 (22.8–26.4)	1.12 (1.03–1.22)	0.008	22.9 (19.3–27.2)	1.29 (1.06–1.57)	0.011
Prior BE diagnosis						
No	25.2 (24.3–26.2)	1		25.3 (23.3–27.4)	1	
Yes	48.0 (41.9–54.9)	0.53 (0.41–0.68)	<0.001	43.7 (33.8–56.5)	0.57 (0.37–0.88)	0.011
Number of endoscopies						
0	25.6 (24.3–27.1)	1		20.9 (16.2–26.8)	1	
1	25.5 (24.3–26.7)	0.91 (0.85–0.97)	0.003	25.9 (23.7–28.2)	0.85 (0.70–1.02)	0.086
≥2	32.4 (28.8–36.5)	0.76 (0.66–0.88)	<0.001	35.2 (28.7–43.2)	0.64 (0.48–0.84)	0.002
Number of years of ≥6 months of PPI or H2RAs						
0	26.0 (25.0–27.2)	1		25.5 (23.2–28.0)	1	
1–3	27.4 (24.8–30.4)	1.03 (0.93–1.14)	0.584	28.7 (23.1–35.9)	1.06 (0.85–1.33)	0.593
4–6	22.6 (19.8–25.9)	1.13 (1.00–1.26)	0.041	25.3 (19.6–32.5)	1.05 (0.82–1.34)	0.715
7–9	26.8 (23.0–31.3)	0.98 (0.83–1.14)	0.764	32.0 (24.5–41.8)	0.84 (0.60–1.17)	0.298
≥10	26.3 (22.5–30.7)	1.00 (0.86–1.17)	0.989	26.5 (18.6–37.6)	1.03 (0.72–1.48)	0.880
Most severe prior upper GI diagnosis						
No record	25.3 (24.1–26.6)	1		25.3 (22.6–28.2)	1	
Indigestion/reflux	25.5 (23.9–27.1)	0.94 (0.88–1.01)	0.172	27.4 (24.1–31.1)	0.92 (0.78–1.08)	0.293
Ulcer	27.1 (19.0–38.5)	1.01 (0.73–1.39)	0.969	33.5 (17.8–63.1)	0.72 (0.32–1.62)	0.426
Oesophagitis	27.8 (23.7–32.6)	0.90 (0.76–1.05)	0.180	30.3 (21.9–42.0)	0.51 (0.29–0.89)	0.018
Hiatus hernia	32.7 (29.3–36.6)	0.78 (0.68–0.90)	<0.001	26.7 (21.1–33.9)	0.98 (0.76–1.26)	0.870
Strictures	24.4 (20.8–28.5)	1.04 (0.91–1.18)	0.613	21.9 (14.9–32.4)	1.11 (0.80–1.56)	0.530
Year of diagnosis						
1993–1999	20.0 (17.8–22.6)	1		27.9 (21.8–35.7)	1	
2000–2006	24.7 (23.2–26.2)	0.75 (0.68–0.82)	<0.001	26.3 (23.6–29.3)	1.05 (0.84–1.32)	0.669
2007–2013	28.1 (26.8–29.4)	0.61 (0.55–0.67)	<0.001	25.9 (23.1–29.1)	1.06 (0.84–1.35)	0.611
Number of primary care consultations within one year prior to diagnosis						
0–7	28.1 (26.6–29.6)	1		26.6 (23.7–29.8)	1	
8–14	25.0 (23.6–26.5)	1.11 (1.03–1.19)	0.007	28.5 (25.3–32.1)	0.99 (0.84–1.17)	0.914
≥15	24.1 (22.4–25.9)	1.23 (1.14–1.33)	<0.001	22.6 (19.2–26.6)	1.39 (1.17–1.66)	<0.001

Abbreviations: BE, Barrett oesophagus; EAC, oesophageal adenocarcinoma; EC, oesophageal cancer; H2RA, H2 receptor antagonist; PPI, proton pump inhibitor.

BMI: Underweight: <18.5; Normal: 18.5–<25; Overweight: 25–<30; Obese ≥30.

^a^ 2‐year relative survival estimated from the multivariate model.

^b^ Multivariate model includes variables age at diagnosis, BMI category, smoking, prior BE diagnosis, number of endoscopies, number of years of ≥6 months of PPI or H2RAs, most severe prior upper GI diagnosis and year of diagnosis.

Results from the multivariate model showed that patients with a prior BE diagnosis had a better prognosis than those with no BE (EHR = 0.53; 95% CI: 0.41–0.68) in the overall sample as well as the EAC subgroup (EHR = 0.57; 0.37–0.88). Furthermore, patients with 2 or more endoscopies prior to the cancer diagnosis had better survival than those who had undergone no endoscopies (EHRs = 0.76; 0.66–0.88) in the overall EC cohort as well as the EAC subgroup (EHRs = 0.64; 0.48–0.84). Table 2 shows that patients diagnosed from 2000 onwards and patients with a prior diagnosis of a hiatus hernia had significantly better survival in the EC cohort; however, this effect was not observed in the EAC subgroup. Patients with more than 15 consultations in the previous year (as an indication of co‐morbidities) had significantly worse survival in both cohorts. Furthermore, current smokers had significantly worse survival than never or ex‐smokers; however, this effect was only observed in the EC cohort. Equivalent results were also observed when each risk factor was separately entered into the survival model (Table S4).

**FIGURE 2:**
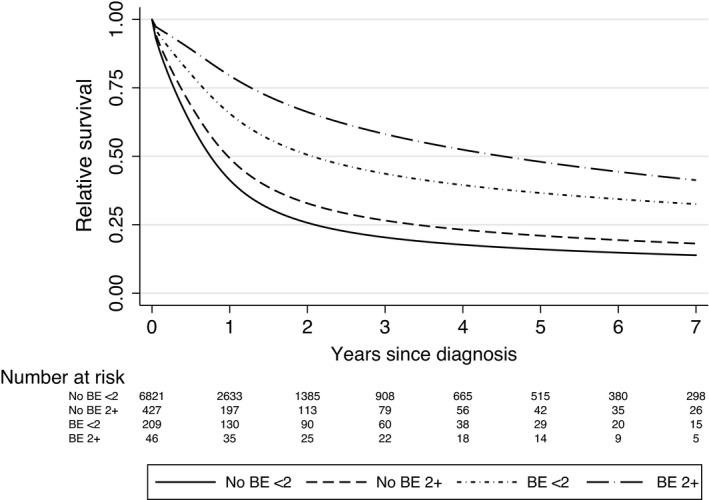
Flexible parametric survival model curves comparing survival for EC with or without prior BE diagnosis stratified by <2 or 2 or more endoscopies adjusted for age and year of diagnosis; No BE, no prior BE diagnosis; BE, prior BE diagnosis; <2, fewer than 2 endoscopies; 2+, 2 or more endoscopies. BE, Barrett oesophagus

Cross‐stratifying by both prior BE (yes/no) and prior endoscopies (none or 1/2 or more) showed that 2‐year survival further differed by endoscopy within each group (Figure 2): amongst patients with prior BE survival was 50.0% (43.6–57.3) for fewer than two endoscopies and 65.4% (52.5–81.6) amongst those with ≥2 endoscopies. In contrast, amongst patients with no prior BE diagnosis 2‐year relative survival was 24.3% (23.3–25.4) for fewer than two and 31.9% (28.0–36.3) for ≥2 endoscopies.

### 3.3 Lead‐time adjustment

**FIGURE 3:**
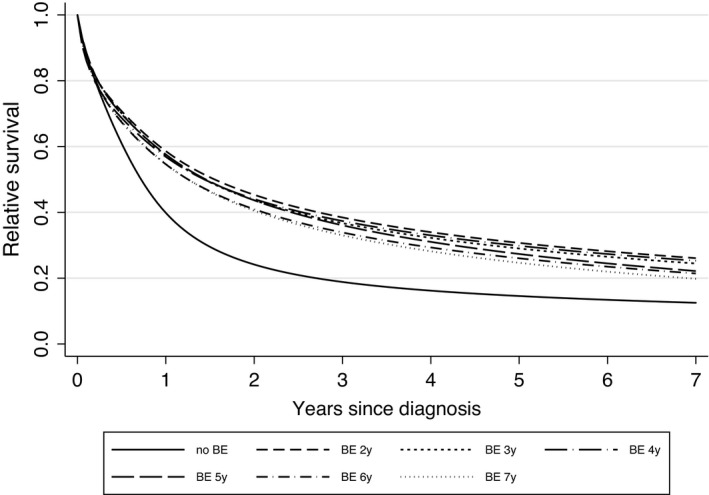
Flexible parametric survival model curves comparing survival for with or without prior BE diagnosis adjusted for lead‐time using a 50% truncated exponential lead‐time with curves for mean lead times ranging from 2 to 7 years indicated as 2y, 3y, …, 7y. Note that the curves are not forced to have proportional hazards. FPMs were also adjusted for age and year of diagnosis. BE, Barrett oesophagus

Using a mixture distribution of lead‐time with 50% having no lead‐time and 50% having a truncated exponential (Figure 3) resulted in a close overlap of the survival curves for the first 6 months regardless of the mean sojourn time used, but still results in a real survival advantage for the BE group. This could, for example, be observed as a 20% absolute difference in 2‐year survival or 10% by 7 years using a mixture exponential with a mean of 5 years in the 50% with a lead‐time. The absolute difference in 2‐year survival ranged from 15% for a 7‐year mixed sojourn time to slightly over 20% for a 2‐year mixed sojourn time, as can be estimated from Figure 3. This range slightly increases with time since diagnosis, to a survival difference of 8% between the lowest and highest lead‐time. Using this method, lead‐time adjusted EHRs ranged from 0.71 (95% CI 0.58–0.87) for a 2‐year mixed sojourn time to 0.77 (95% CI 0.63–0.95) for a 7‐year mixed sojourn time.

## 4 DISCUSSION

### 4.1 Summary of main findings

Within our large UK cohort of newly diagnosed EC patients and a subgroup of EAC patients only a small percentage, 3.4% of all EC and 5.3% of EAC patients, were diagnosed with BE at least 6 months prior to their cancer diagnosis. Our survival analysis showed that cancer patients with a prior BE diagnosis had a better prognosis than those without BE. A prior BE diagnosis conferred a survival benefit even if no or only one surveillance endoscopy had been carried out. This survival benefit was attenuated when adjusting for lead‐time bias, but patients with a prior BE diagnosis continued to have a better prognosis up to 7 years post‐diagnosis.

### 4.2 Interpretation

We observed that a prior BE diagnosis even without regular surveillance endoscopy (one or no recorded endoscopies) resulted in better survival compared to patients without prior BE diagnosis. This could either be due to patients with prior BE being more likely to consult with their GP if their symptoms worsen, or to be referred to secondary care.^25^ Alternatively, Sawas and colleagues recently found that EACs with coexisting BE at the time of cancer diagnosis but without surveillance had better survival compared to EAC without BE, even when adjusting for tumour stage and treatment.^12^ They suggested that carcinogenesis of these two different cancers could occur via two different molecular sequences resulting in two phenotypically different types of EAC, one with histologically identifiable BE and one without. We could therefore hypothesise that the better survival of patients with BE without regular surveillance compared to patients without a prior BE diagnosis in our cohort could also be explained by these two phenotypes with differing prognosis.

We initially carried out lead‐time adjustment based on methodology developed by Duffy and colleagues for screen‐detected breast cancers.^24^ However, the EC patients in this study did not have screen‐detected cancers as usually defined. Instead, some of them would have had surveillance for existing BE and hence might have had their cancers diagnosed earlier. We, therefore, used a mixed distribution for lead‐time with 50% having no lead‐time and 50% having a truncated exponential lead‐time using a range of means from 2 to 7 years. Using this method, we observed a close overlap in survival for the first 6 months. After this, the survival benefit observed for patients with prior BE was attenuated from an absolute difference in 2‐year survival of 25% (Figure 1) to a range from slightly over 20% for a 2‐year to 15% for a 7‐year mixed lead‐time (Figure 3). The survival benefit was still observable though, as can also be seen in lead‐time adjusted EHRs ranging from 0.71 for 2‐year mixed to 0.77 for 7‐year mixed lead‐times.

### 4.3 Context of other literature

A systematic review and meta‐analysis of 12 cohort studies on the effect of surveillance in patients with BE found that patients with surveillance‐detected EAC had lower EAC‐related and all‐cause mortality with a HR of 0.59 (95% CI 0.45–0.76),^26^ very similar to the excess mortality we estimated. They furthermore found that surveillance was associated with the detection of EAC at an earlier stage (RR = 2.11 for diagnosis at stage 0 or 1 in the surveillance group). Two studies found that EAC patients with a prior BE diagnosis had significantly better survival, with similar adjusted HRs to the ones we observed: 0.44 for a cohort of EAC patients from Northern Ireland and 0.51 for a cohort of EAC patients identified from the Veterans Affairs Central Cancer Registry.^27^, ^28^ Three cohort studies of EAC patients identified from the SEER‐Medicare database also found that a prior BE diagnosis was associated with better survival with adjusted HRs ranging from 0.45 to 0.72. However, the Medicare database only includes patients from age 65 upwards, resulting in older cohorts than the one we analysed.^29^, ^30^, ^31^ As 5‐year survival decreases with age we would expect worse survival of these patients compared to our cohort. Four of the studies included in the systematic review carried out lead‐time adjustment,^28^, ^32^, ^33^, ^34^ which either attenuated or eliminated the observed benefit depending on the length of sojourn time. Even though these studies also corrected for lead‐time bias, the adjustments differed. Firstly, this methodology depends on the mean sojourn time.^35^ In the absence of a reliable estimate of the sojourn time for EC several studies used differences in mean age at cancer diagnosis between prior and no prior BE groups as an estimate of lead‐time,^27^, ^30^, ^32^ but earlier age at diagnosis could also be due to a number of risk factors. Secondly, the majority of these studies investigated the impact of a prior BE diagnosis and not regular surveillance for BE. Only a proportion of these cancers would have been diagnosed by surveillance endoscopy and thus be subject to lead‐time bias. In our mixed distribution for lead‐time only 50% of individuals were therefore getting adjusted for lead‐time bias. The only study that compared EAC survival by whether the cancer was diagnosed by surveillance or non‐surveillance endoscopy, by El‐Serag et al.,^32^ did not observe any impact of lead‐time bias on the survival benefit of surveillance endoscopy.

### 4.4 Limitations and strength

Firstly, GP practices in CPRD do not represent a random sample of all UK practices with population coverage ranging between 1.6 and 13.6% for different UK regions.^13^ However, it overall covers 6.9% of the UK population and patients are broadly representative of the general population with regards to age, sex and ethnicity. Secondly, data is entered as part of a GP consultation and not for the purpose of research. Only records using Read codes are available via the CPRD whereas free text or scanned documents are not, resulting in potentially missing information.^36^ There is particularly a risk that details about hospital admission or procedures, like endoscopies, are missed if these are not entered into the patient record, though our use of HES data mitigates this. Overall, linked data was available for 59% of our EC cohort, allowing us to identify EAC patients. All BE patients in this subgroup had at least one record of an endoscopy prior to their cancer diagnosis (Table S3) confirming that endoscopy records for patients with linkages were more complete. We also used HES to identify any additional BE diagnosis not recorded in the CPRD dataset. A BE specific ICD10 code has only been available since 2006 so any BE diagnosis not recorded in CPRD before 2006 would have been missed. Missing BE diagnosis codes would result in misclassification of BE patients as ‘no BE’ leading to classification bias. This could substantially decrease the survival benefit seen for BE patients. Only 3.4% of all EC patients had a record of a prior BE diagnosis. This being such a small proportion, these patients could be different in a number of ways from the rest of the cohort. However, this EC cohort and the group of patients with a prior BE diagnosis represent the entire population in the CPRD cohort, which makes the comparison clinically valid. An effective BE surveillance strategy should result in a stage shift towards early‐stage EC detection. As we only had stage and grade data available for a small number of patients, we could not study this directly. Cause of death was only available for patients with linked data and is often not coded accurately. Therefore, instead of using cause specific mortality, we estimated relative survival. We did not carry out a sensitivity analysis comparing patients with concurrent BE/EC diagnosis with no BE to address the question of biological differences between tumours with and without BE, as, firstly, ECs with concurrent BE are thought to be more likely to be earlier stage and, secondly, we did not believe that these BE diagnoses would have been systematically recorded.

## 5 CONCLUSION

In conclusion, we found that only a very small proportion of EC and EAC patients had a previous diagnosis of BE. Patients with a prior diagnosis had a survival advantage compared to those without. This was not explained solely by surveillance endoscopies, which warrants further research. These findings support the continuation of surveillance of BE patients, as well as the importance of identifying more BE cases in high‐risk populations.

## CONFLICT OF INTERESTS

The authors declare that they have no competing interests.

## AUTHOR CONTRIBUTIONS

Study concept and design: JO, FP, RF, WH, PS; acquisition of data: JO; analysis and interpretation of data: FP, JO, PS; drafting of the manuscript: JO, FP; critical revision of the manuscript for important intellectual content: JO, FP, PS, RF, WH; statistical analysis: FP, PS; funding acquisition: PS. The sponsor had no involvement in the study.

## ETHICS

The study was approved by the CPRD Independent Scientific Advisory Committee (ISAC) (protocol number 15_026).

## Supporting information

Supplementary Material

## Data Availability

The data that support the findings of this study were obtained from the Clinical Practice Research Datalink (CPRD) under license from the UK Medicines and Healthcare products Regulatory Agency. The authors can provide data specifications for any data requests to the Independent Scientific Advisory Committee (ISAC). We thank the Clinical Practice Research Datalink (CPRD) for the provision of the data used in this study.
