# Supplementary material for: Impact of Barrett oesophagus diagnoses and endoscopies on oesophageal cancer survival in the UK: A cohort study
Source: Cancer Med. 2021 Dec 16;11(4):1160–71. doi: 10.1002/cam4.4484 (PMC8855914; doi:10.1002/cam4.4484)
Supplement: Supplementary file 1 — Supplementary Material [file CAM4-11-1160-s001.docx]

**SUPPLEMENTARY MATERIALS**

**Section 1 (Methods): Study cohort selection**

The following Read codes were provided to the CPRD for the extraction of the initial study cohort (Supplementary Table 3). As we were originally hoping to include Barrett’s esophagus (BE) with high grade dysplasia and carcinomas of the gastro-esophageal junction, we included all begin neoplasms and carcinomas *in situ* of the esophagus and malignant cancers of the stomach in this list. However, after the data was obtained and examined we applied additional exclusion criteria. Only Read codes in bold were included in the final dataset.

Further eligibility criteria applied by CPRD

- Age 50 or older
- Cancer occurred in the study period: 01/01/1987 – 31/12/2014 (1^st^ study period)
- At least 3 years CPRD registration prior to EC diagnosis

Further exclusions applied after receipt of the initial data from CPRD.

Firstly, the study period was shortened to 01/01/1993 to 31/12/2013 (2^nd^ study period) due to data scarcity prior to 1993 and incompleteness after 2013. Secondly, it was not possible to identify cancers of the gastro-esophageal junction related to acid reflux using cancer registry data, so all cancers of the stomach were excluded. Furthermore, records on carcinomas *in situ* and benign cancers were incomplete, so only malignant neoplasms (B100.00 to B107.00, B10y.00, B10z.00) were included in the final cohort. It was assumed that non-specific records of esophageal cancer (B10z.11) would be records of malignant cancers as these occur most frequently. Lastly, any cases included by CPRD but which did not fit the criteria were excluded.

**Study Design**

Use of different data sources

Data from clinical events that occur outside of primary care, like for example deaths or diagnoses from tests carried out in secondary care, require GPs receiving this information and coding it in their clinic database in a timely manner. To ensure that the most complete data was used for each variable, we combined CPRD data with additional sources, like Office of National Statistics (ONS) for deaths and National Cancer Registration and Analysis Service (NCRAS) for cancer data. We used linked NCRAS and Hospital Episode Statistics (HES) data to fill in missingness, for example to obtain endoscopy records, which had not been coded in CPRD.

For deaths, as ONS date of death is always directly based on the death certificates, date of death was obtained from ONS, where ONS data was available. For patients without linked ONS data the date of death recorded in CPRD was used. A previous study investigating date of death recording in CPRD with ONS found that 98% of ONS deaths were also identified in CPRD, with exact agreement on the death date between CPRD and ONS in 70% of deaths between 1998 and 2013.(1) When we previously compared a larger CPRD cohort of over 10,000 EC and stomach cancers all with ONS linkages, we observed more than 90% overlap between CPRD and ONS records. Comparing date of death in the EC cohort in this study we observed differences in the date recorded between CPRD and ONS files for 729 patients, however, for 90% of these records the dates differed by 28 days or less.

**Supplementary Table 1: Esophageal and stomach cancer specific Medical and Read codes. Read codes used for the final dataset are highlighted in bold.**

| Medical code | Read code | Description | Initial CPRD cohort | Final study cohort |
| --- | --- | --- | --- | --- |
| CANCER OF THE ESOPHAGUS | | | ✓ | ✓ |
| 1062 | **B10..00** | **Malignant neoplasm of esophagus** | ✓ | ✓ |
| 61695 | **B100.00** | **Malignant neoplasm of cervical esophagus** | ✓ | ✓ |
| 41362 | **B101.00** | **Malignant neoplasm of thoracic esophagus** | ✓ | ✓ |
| 63470 | **B102.00** | **Malignant neoplasm of abdominal esophagus** | ✓ | ✓ |
| 50789 | **B103.00** | **Malignant neoplasm of upper third of esophagus** | ✓ | ✓ |
| 54171 | **B104.00** | **Malignant neoplasm of middle third of esophagus** | ✓ | ✓ |
| 42416 | **B105.00** | **Malignant neoplasm of lower third of esophagus** | ✓ | ✓ |
| 67497 | **B106.00** | **Malignant neoplasm, overlapping lesion of esophagus** | ✓ | ✓ |
| 98142 | **B107.00** | **Siewert type I adenocarcinoma** | ✓ | ✓ |
| 53591 | **B10y.00** | **Malignant neoplasm of other specified part of esophagus** | ✓ | ✓ |
| 30700 | **B10z.00** | **Malignant neoplasm of esophagus NOS** | ✓ | ✓ |
| 4865 | **B10z.11** | **Esophageal cancer** | ✓ | ✓ |
| 16077 | B710.00 | Benign neoplasm of esophagus | ✓ |  |
| 3623 | B710.11 | Benign esophageal polyp | ✓ |  |
| 59937 | B710100 | Benign neoplasm of upper 1/3 of esophagus | ✓ |  |
| 56870 | B710300 | Benign neoplasm of lower 1/3 of esophagus | ✓ |  |
| 72603 | B710z00 | Benign neoplasm of esophagus NOS | ✓ |  |
| 8244 | B801.00 | Carcinoma in situ of esophagus | ✓ |  |
| 99155 | B801000 | Carcinoma in situ of upper 1/3 esophagus | ✓ |  |
| 64274 | B801100 | Carcinoma in situ of middle 1/3 esophagus | ✓ |  |
| 56077 | B801200 | Carcinoma in situ of lower 1/3 esophagus | ✓ |  |
| 44228 | B801z00 | Carcinoma in situ of esophagus NOS | ✓ |  |
| 25215 | 1J0D.00 | Suspected upper gastrointestinal cancer | ✓ |  |
| 20570 | 14CB.00 | H/O Upper GIT Neoplasm | ✓ |  |
| CANCER OF THE STOMACH | | |  |  |
| 32022 | B110.00 | Malignant neoplasm of cardia of stomach | ✓ |  |
| 100584 | B110000 | Malignant neoplasm of cardiac orifice of stomach | ✓ |  |
| 22894 | B110100 | Malignant neoplasm of cardio-esophageal junction of stomach | ✓ |  |
| 94278 | B110111 | Malignant neoplasm of gastro-esophageal junction | ✓ |  |
| 37859 | B110z00 | Malignant neoplasm of cardia of stomach NOS | ✓ |  |
| 65372 | B11yz00 | Malignant neoplasm of other specified site of stomach NOS | ✓ |  |
| 14800 | B11z.00 | Malignant neoplasm of stomach NOS | ✓ |  |

**Supplementary Table 2: Barrett’s esophagus specific Medical and Read codes**

| MEDICAL CODE | READ CODE | DESCRIPTION |
| --- | --- | --- |
| 4614 | J101611 | Barrett's esophagus |
| 5596 | J102500 | Barrett's ulcer of esophagus |
| 99526 | J10y600 | Barrett's esophagus |

**Section 2 (Results):**

**Supplementary Figure 1: Strobe flow diagram of study cohort selection**

Eligible patients with Read codes during 1^st^ study period identified by CPRD (Supp Table 1)

n=24,276

Exclusions applied by CPRD:

- < 50 years: n=1463
- < 36 months follow – up prior to diagnosis: n=4890

Included in study cohort by CPRD: n=17,923

Included in final study cohort (Supp Table 1): n=7,503

Exclusions applied after data examination:

- Outside 2^nd^ study period: n=1,463
- Non malignant cancers: n=8,329
- <50 years: n=2
- Exit date prior to or on entry date: n=61

Primary care data

Linked data

Linkages available for 59% of EC patients: n=3,920

BE: n=255

No BE: n=7,248

**Supplementary Table 3**. Characteristics of esophageal adenocarcinoma (EAC) patients according to prior Barrett's esophagus (BE) diagnosis

|  |  | **all EAC** | **EAC without prior BE diagnosis** | **EAC with prior BE diagnosis** |
| --- | --- | --- | --- | --- |
|  |  | **n=1476** | **n=1398** | **n=78** |
| **Demographic characteristics** | |  |  |  |
| **Age at cancer diagnosis (years),** | | | | |
|  | **Mean (SD)** | 71 (10) | 71 (10) | 72 (10) |
|  | **Median (IQR)** | 72 (15) | 71 (15) | 74 (13) |
| **Sex, n (%)** | |  |  |  |
|  | **Male** | 1148 (78) | 1084 (76) | 64 (82) |
|  | **Female** | 328 (22) | 314 (22) | 14 (18) |
| **BMI, n (%)** | |  |  |  |
|  | **Underweight** | 61 (4) | 55 (4) | 6 (8) |
|  | **Normal** | 432 (29) | 404 (29) | 28 (36) |
|  | **Overweight** | 330 (22) | 307 (22) | 23 (30) |
|  | **Obese** | 166 (11) | 162 (12) | 4 (5) |
|  | **Missing** | 487 (33) | 470 (34) | 17 (22) |
| **Smoking, n (%)** | |  |  |  |
|  | **Current** | 348 (24) | 333 (26) | 15 (19) |
|  | **Never** | 625 (42) | 581 (42) | 44 (56) |
|  | **Ex** | 393 (27) | 375 (27) | 18 (22) |
|  | **Missing** | 110 (7) | 109 (8) | 1 (1) |
| **IMD categories, n (%)** | |  |  |  |
|  | **1** | 248 (17) | 233 (17) | 15 (19) |
|  | **2** | 291 (20) | 278 (20) | 13 (17) |
|  | **3** | 318 (22) | 304 (22) | 14 (18) |
|  | **4** | 320 (22) | 305 (22) | 15 (19) |
|  | **5** | 299 (20) | 278 (20) | 21 (27) |
| **Clinical characteristics** | |  |  |  |
| **Prior upper GI diagnosis, n (%)** | | 741 (50) | 675 (48) | 66 (85) |
| **Prior endoscopies, n (%)** | |  |  |  |
|  | **none** | 158 (11) | 158 (11) | 0 (0) |
|  | **1** | 1178 (80) | 1126 (81) | 52 (67) |
|  | **2+** | 140 (9) | 114 (8) | 26 (33) |
| **PPI / H2RA presciption patterns, n (%)** | | | | |
|  | **1 prescription<28 days** | 5 (0.3) | 5 (0.4) | 0 (0) |
|  | **2+ prescriptions in 1 year** | 79 (5) | 79 (6) | 0 (0) |
|  | **no prescriptions** | 401 (27) | 401 (29) | 0 (0) |
|  | **1 prescription in 1 year** | 262 (18) | 262 (19) | 0 (0) |
|  | **2 prescriptions in 6 months** | 280 (19) | 278 (20) | 2 (3) |
|  | **6 months in 1 year** | 449 (30) | 373 (27) | 76 (97) |
| **Number of years with 6+ PPI / H2RA, n (%)** | | | | |
|  | **0** | 1027 (70) | 1025 (73) | 2 (3) |
|  | **1-3** | 147 (10) | 137 (10) | 10 (13) |
|  | **4-6** | 127 (9) | 110 (8) | 17 (22) |
|  | **7-9** | 92 (6) | 72 (5) | 20 (26) |
|  | **10+** | 83 (5) | 54 (4) | 29 (37) |
| **Most severe upper GI diagnosis prior to cancer diagnosis, n (%)** | | | |  |
|  | **No prior diagnosis** | 735 (50) | 723 (52) | 12 (15) |
|  | **Indigestion/reflux** | 454 (31) | 432 (31) | 22 (28) |
|  | **Ulcer** | 12 (1) | 10 (1) | 2 (3) |
|  | **Esophagitis** | 66 (4) | 56 (4) | 10 (13) |
|  | **Hiatus hernia** | 148 (10) | 120 (9) | 28 (36) |
|  | **Strictures** | 61 (4) | 57 (4) | 4 (5) |
| **Year of diagnosis, n (%)** | |  |  |  |
|  | **1993-1999** | 119 (8) | 118 (8) | 1 (2) |
|  | **2000-2006** | 734 (50) | 697 (50) | 37 (47) |
|  | **2007-2013** | 623 (42) | 583 (42) | 40 (51) |
| **Number of consultation within one year prior to diagnosis, n (%)** | | | |  |
|  | **0-7** | 595 (40) | 566 (40) | 29 (37) |
|  | **8-14** | 512 (35) | 484 (35) | 28 (36) |
|  | **≥15** | 3369 (25) | 348 (25) | 21 (26) |
|  |  |  |  |  |

**Supplementary table 4.** Flexible parametric excess hazard ratios (EHR) for relative survival adjusted for age and year of diagnosis for all EC and EAC patients

|  |  | **EC** | | **EAC** | |
| --- | --- | --- | --- | --- | --- |
| **Characteristic** | | **EHR adjusted for age and year of diagnosis (95% CI)** | | **EHR adjusted for age and year of diagnosis (95% CI)** | |
|  |  | **EHR** | **P value** | **EHR** | **P value** |
| **Age at diagnosis** | |  |  |  |  |
|  | **<65** | 1 |  | 1 |  |
|  | **65-69** | 1.22 (1.10 - 1.37) | < 0.001 | 1.27 (1.00 - 1.60) | 0.049 |
|  | **70-74** | 1.32 (1.19 - 1.47) | < 0.001 | 1.41 (1.12 - 1.77) | 0.003 |
|  | **75-79** | 1.53 (1.38 - 1.69) | < 0.001 | 1.64 (1.32 - 2.05) | < 0.001 |
|  | **80-84** | 1.84 (1.66 - 2.04) | < 0.001 | 1.91 (1.51 - 2.42) | < 0.001 |
|  | **85-90** | 2.56 (2.28 - 2.87) | < 0.001 | 2.46 (1.89 - 3.19) | < 0.001 |
|  | **>90** | 2.76 (2.37 - 3.21) | < 0.001 | 2.72 (1.83 - 4.04) | < 0.001 |
| **Sex** |  |  |  |  |  |
|  | **Male** | 1 |  | 1 |  |
|  | **Female** | 0.97 (0.91 - 1.03) | 0.38 | 1.08 (0.94 - 1.25) | 0.270 |
| **BMI category** | |  |  |  |  |
|  | **Underweight** | 1.12 (0.99 - 1.27) | 0.081 | 0.85 (0.62 - 1.17) | 0.331 |
|  | **Normal** | 1 |  | 1 |  |
|  | **Overweight/obese** | 1.14 (1.05 - 1.23) | 0.001 | 1.36 (1.18 - 1.58) | < 0.001 |
|  | **Missing** | 1.21 (1.12 - 1.30) | < 0.001 | 1.32 (1.14 - 1.53) | < 0.001 |
| **Smoking** | |  |  |  |  |
|  | **Current** | 1.28 (1.18 - 1.38) | < 0.001 | 1.11 (0.95 - 1.29) | 0.194 |
|  | **Never** | 1 |  | 1 |  |
|  | **Ex** | 1.00 (0.92 - 1.09) | 0.96 | 1.02 (0.88 - 1.18) | 0.798 |
|  | **Missing** | 1.67 (1.50 - 1.85) | < 0.001 | 1.97 (1.57 - 2.46) | < 0.001 |
| **IMD categories** | |  |  |  |  |
|  | **1** | 1 |  | 1 |  |
|  | **2** | 1.06 (0.97 - 1.16) | 0.212 | 1.15 (0.94 - 1.39) | 0.168 |
|  | **3** | 1.07 (0.99 - 1.17) | 0.104 | 1.15 (0.95 - 1.40) | 0.138 |
|  | **4** | 1.08 (0.99 - 1.17) | 0.077 | 1.19 (0.99 - 1.44) | 0.068 |
|  | **5** | 1.11 (1.02 - 1.21) | 0.014 | 1.21 (1.00 - 1.46) | 0.054 |
| **Prior BE diagnosis** | |  |  |  |  |
|  | **No** | 1 |  | 1 |  |
|  | **Yes** | 0.47 (0.37 - 0.60) | < 0.001 | 0.47 (0.31 - 0.71) | < 0.001 |
| **Number of endoscopies** | |  |  |  |  |
|  | **0** | 1 |  | 1 |  |
|  | **1** | 0.91 (0.85 - 0.96) | 0.002 | 0.88 (0.71 - 1.09) | 0.246 |
|  | **≥2** | 0.73 (0.63 - 0.84) | < 0.001 | 0.62 (0.45 - 0.85) | 0.003 |
| **Number of years of ≥6 months of PPI or H2RAs** | | |  |  |  |
|  | **0** | 1 |  | 1 |  |
|  | **1-3** | 1.01 (0.92 - 1.11) | 0.824 | 1.11 (0.89 - 1.38) | 0.35 |
|  | **4-6** | 1.05 (0.94 - 1.17) | 0.395 | 0.94 (0.74 - 1.20) | 0.613 |
|  | **7-9** | 0.84 (0.72 - 0.98) | 0.03 | 0.71 (0.51 - 0.97) | 0.033 |
|  | **≥10** | 0.81 (0.70 - 0.94) | 0.004 | 0.76 (0.55 - 1.06) | 0.102 |
| **Most severe prior upper GI diagnosis** | | |  |  |  |
|  | **No recorded symptoms** | 1 |  | 1 |  |
|  | **Indigestion / reflux** | 0.94 (0.88 - 1.01) | 0.092 | 0.91 (0.78 - 1.07) | 0.263 |
|  | **Ulcer** | 1.01 (0.73 - 1.39) | 0.963 | 0.81 (0.36 - 1.81) | 0.607 |
|  | **Esophagitis** | 0.85 (0.73 - 1.00) | 0.047 | 0.45 (0.26 - 0.78) | 0.004 |
|  | **Hiatus hernia** | 0.71 (0.62 - 0.81) | < 0.001 | 0.83 (0.65 - 1.05) | 0.119 |
|  | **Strictures** | 1.01 (0.88 - 1.15) | 0.885 | 1.04 (0.74 - 1.46) | 0.813 |
| **Year of diagnosis** | |  |  |  |  |
|  | **1993-1999** | 1 |  | 1 |  |
|  | **2000-2006** | 0.66 (0.60 - 0.72) | < 0.001 | 0.97 (0.75 - 1.25) | 0.808 |
|  | **2007-2013** | 0.50 (0.46 - 0.55) | < 0.001 | 0.86 (0.67 - 1.12) | 0.27 |
| **Number of consultation within one year prior to diagnosis** | | | |  |  |
|  | **0-7** | 1 |  | 1 |  |
|  | **8-14** | 1.07 (1.02 - 1.15) | 0.055 | 0.97 (0.82 - 1.15) | 0.746 |
|  | **≥15** | 1.19 (1.10 - 1.29) | < 0.001 | 1.34 (1.12 - 1.59) | 0.001 |
|  |  |  |  |  |  |

*EHR, excess hazard ratios

BMI: Underweight: <18.5; Normal: 18.5 - <25; Overweight: 25 - <30; Obese ³30. PPI, proton pump inhibitor; H2RA, H2 receptor antagonist.

**References**

1. Gallagher AM, Dedman D, Padmanabhan S, Leufkens HGM, de Vries F. The accuracy of date of death recording in the Clinical Practice Research Datalink GOLD database in England compared with the Office for National Statistics death registrations. Pharmacoepidemiology and drug safety. 2019;28(5):563-9.
